# Psychometric properties of screening questionnaires to detect depression in primary healthcare setting in rural Ethiopia

**DOI:** 10.1186/s12875-022-01755-2

**Published:** 2022-06-02

**Authors:** Kassahun Habtamu, Rahel Birhane, Girmay Medhin, Charlotte Hanlon, Abebaw Fekadu

**Affiliations:** 1 School of Psychology, College of Education and Behavioral Studies, Addis Ababa University, P.O.BOX: 1176, Addis Ababa, Ethiopia; 2 Department of Psychiatry, School of Medicine, College of Health Sciences, Addis Ababa University, Addis Ababa, Ethiopia; 3 Aklilu Lemma Institute of Pathobiology, Addis Ababa University, Addis Ababa, Ethiopia; 4 Centre for Global Mental Health, Health Service and Population Research Department, and WHO Collaborating Centre for Mental Health Research and Training, Institute of Psychiatry, Psychology and Neuroscience, King’s College London, London, UK; 5 Centre for Innovative Drug Development and Therapeutic Trials for Africa (CDT-Africa), Addis Ababa University, Addis Ababa, Ethiopia; 6 Global Health & Infection Department, Brighton and Sussex Medical School, Brighton, UK; 7 Center for Affective Disorders, Department of Psychological Medicine, Institute of Psychiatry, Psychology and Neuroscience, King’s College London, London, UK

**Keywords:** Depression, Patient health questionnaire, Primary healthcare, Psychometric properties, Convergent validity, Construct validity, Screening, Rural Ethiopia

## Abstract

**Background:**

Much of the research about the validity of depression screening questionnaires is on criterion validity. Evidence is scarce on the concurrent, convergent and construct validity of these measures, particularly from low-income countries. This study aimed to evaluate the psychometric properties of depression screening questionnaires in primary healthcare (PHC) in rural Ethiopia.

**Methods:**

A facility-based cross-sectional study was conducted with 587 participants recruited from patients attending three PHC facilities and two ‘Holy water sites’ (places where religious treatment is being provided). The psychometric properties of five mental health screening questionnaires were evaluated: the nine item Patient Health Questionnaire (PHQ-9), the two item version of PHQ-9 (PHQ-2), a version of PHQ-9 with two added items of irritability and noise intolerance (PHQ-11), the Patient Health Questionnaire-15 (PHQ-15), and the World Health Organization-Five Well-being Index (WHO-5). Clinical diagnosis of depression was ascertained by psychiatrists. We analyzed data using exploratory factor analysis, Spearman’s rank order correlation coefficient (Rho), the Mann Whitney test of the equality of medians, univariate logistic regression and Cronbach’s alpha.

**Results:**

PHQ-9, PHQ-11 and WHO-5 were found to be unidimensional, with items in each scale highly loading onto one factor (factor loadings ranging from 0.64 to 0.87). The items of each instrument were internally consistent, with Cronbach’s alpha ranging from 0.72 (PHQ-2) to 0.89 (PHQ-11). Scores for all screening scales were moderately or highly correlated with each other (Rho = 0.58 to 0.98) and moderately correlated with anxiety and disability scores. Median scores of all screening scales were significantly higher in those diagnosed with depression. The association of items measuring emotional and cognitive symptoms with the diagnosis of depression was stronger than the association with items measuring somatic symptoms. Irritability and noise intolerance had higher association with depression diagnosis than PHQ-9 items.

**Conclusion:**

Emotional and cognitive symptoms are more useful than somatic symptoms to predict the diagnosis of depression in the PHC context in Ethiopia. Future research should focus on testing the unidimensionality of PHQ-9, PHQ-11 and WHO-5 using confirmatory factor analysis; establishing the criterion validity of PHQ-11 and WHO-5; and on assessing test-retest reliability of all the measures.

## Background

Depression is the leading cause of disability, as measured by Years Lived with Disability [1], and projected to become the second leading contributor to the global burden of disease by 2030 [2]. Depression is associated with increased use of healthcare resources [3] and results in an enormous economic burden [4] as it is one of the most commonly occurring illnesses and leads to substantial loss in productivity. Depression is associated with elevated morbidity and mortality related to suicide [5]; it is often co-morbid with other physical as well as mental health conditions [6].

The prevalence of depression in primary healthcare (PHC) is higher than in the general population [7]. Around 10% of all primary care visits are depression related and most patients who have depression get treatment by PHC clinicians [8]. As it is indicated in the Federal Ministry of Health’s (FMOH) National Mental Health Strategy [9], depression is one of the priority mental disorders in the PHC setting in Ethiopia. PHC clinicians in Ethiopia followed the mhGAP guideline [10] to treat depression, which follows a stepped care approach. Mild forms of depression are usually addressed with simple psycho-education and psychological counseling as well as monitoring. More severe forms of depression (such as major depressive disorder) are treated with antidepressants. When available selective serotonin reuptake inhibitors are preferred first line treatments; otherwise, tricyclic antidepressants would be prescribed. Additional supportive counseling would also be provided. Specialist centers follow treatment approaches concordant with guidelines, such as the British Association for Psychopharmacology or the American Psychiatric Association’s guideline.

Despite a high prevalence of depression in primary care [7], underrecognition is a major challenge globally [11]. In high-income countries, approximately 50% of primary care doctors correctly identify individuals with depression, and only 34% record it in their notes [12]. In low and middle-income countries (LMICs), the detection of depression in the PHC setting is extremely low. For instance, a study conducted in Ethiopia showed that less than 5% of patients presenting to primary healthcare with potential depression received a clinical diagnosis of depression [8]. Another study conducted in a health center in Malawi found a 0 % depression detection rate by primary healthcare workers [13]. Low levels of detection of depression are jeopardizing the impact of efforts to scale up integration of mental healthcare into PHC [8].

Screening with self-reported questionnaires is considered to be a potentially useful approach to aid PHC clinicians in recognizing patients who may have depression [14]. Guidelines developed in some high income countries recommend routine screening for depression in PHC [15]. A systematic review found that screening questionnaires are likely to be effective in improving recognition of depression when they are used in conjunction with other interventions [16]. However, screening alone does not seem to be effective; it has to be accompanied by disclosure of screening results to the clinician, training of the clinical and other relevant staff, supportive supervision and clear referral pathways [17, 18]. Nevertheless, studies on the utility of screening questionnaires to increase detection of depression in real-world settings are mostly from high-income countries [19] and evidence is scarce whether these instruments are useful in LMICs [16]. In low-income countries, depression screening tools may have effect to improve PHC providers’ diagnosis of depression [20]; and this needs the use of brief and psychometrically sound screening instruments.

A number of brief screening questionnaires that can be used to screen for depression in the PHC setting do exist [16, 19]. However, the bulk of evidence regarding the validity and reliability of these questionnaires is from high income countries [21]. There are some studies on the validity of depression screening tools from LMICs, but much of the evidence is on criterion validity [22]. There is only limited research on the convergent and construct validity of depression screening tools, particularly in low-income country settings. There are a few studies conducted in Ethiopia on the criterion validity of depression screening tools, particularly on the nine item Patient Health Questionnaire (PHQ-9) [20, 23–25]. These studies found that the PHQ-9 has acceptable sensitivity, specificity, positive predictive value and negative predictive value. Only one of these validation studies was conducted in the PHC setting and validated more than one depression screening tool [20]. This study found that PHQ-9, Self Reporting Questionnaire (SRQ-20), and six items and ten items versions of the Kessler Psychological Distress Scale (K6 and K10), but not PHQ-2, had good performance in terms of sensitivity, specificity, positive predictive value and negative predictive value. Almost all of the studies focused on criterion validity of the tools against clinician diagnosis of depression. None of the studies investigated the factor structure of the screening tools and which of the symptoms or items in the scales are associated with diagnosis of depression ascertained by psychiatrists.

This study, therefore, aimed to evaluate the concurrent, convergent and construct validity of depression screening questionnaires in PHC setting in rural Ethiopia. Specifically, we sought to determine i) the factor structure of depression screening questionnaires using exploratory factor analysis ii) convergent and concurrent validity of the instruments by computing the correlation of aggregate scores of each screening tool with other depression measures and measures of other variables which are theoretically known to have correlation with depressive symptoms iii) known group differences by comparing median depressive symptom scores between those who have psychiatrist diagnosis of depression and those who do not have depression diagnosis iv) association of each item of the depression screening tools with psychiatrist depression diagnosis.

## Methods

### Study design

A facility-based cross-sectional study was conducted to investigate the psychometric properties of brief depression screening questionnaires when they are used in PHC setting in rural Ethiopia.

### Study setting and context

The study was conducted in Sodo district, Gurage Zone, Southern Nations, Nationalities and Peoples Region (SNNPR). The district is predominantly rural, and is located 100 km south of Addis Ababa, the capital city of Ethiopia. The population of the district at the time of the study was estimated to be 161,952 (79,356 men and 82,596 women) living in 58 sub-districts [26]. The largest ethnic group in the district is Sodo Gurage (85.3%), Amharic is the official language [8] and 97% of the population are followers of Orthodox Christian [26].

The district has one primary hospital, 8 health centers and 58 health posts [27]. Staff in health centers constituted nurses, health officers, and midwives, who are trained at degree or diploma level. Health centers provide primary care for about 20,000 (rural areas) to 40,000 (urban areas) people; whereas each health post serves 3000–5000 people. Healthcare providers in the health centers and health posts deliver services such as diagnosis and treatment of communicable diseases (e.g. malaria, tuberculosis, and water-borne diseases), family planning, antenatal care, malaria prevention, and give advice on the effects of harmful traditional practices and sanitation. At the time of this study, efforts had been made to integrate mental health services into the primary care level as part of the PRogramme for Improving Mental Healthcare (PRIME) project [28]. Before the start of the PRIME project, people with mental disorders had to travel to an outpatient clinic in Butajira town, which is led by psychiatric nurses; for inpatient psychiatric treatment or interventions for substance use disorders, they had to travel to Addis Ababa [26].

This study was carried out as part of the Improving Detection of depression in primary care in Sub-Saharan Africa (IDEAS) project [29]. The IDEAS study aimed to develop and evaluate interventions that would help improve the recognition of depression in the PHC setting in Ethiopia. To develop this paper, we used the baseline data collected for the IDEAS cohort study.

### Participants and recruitment

**Fig. 1:**
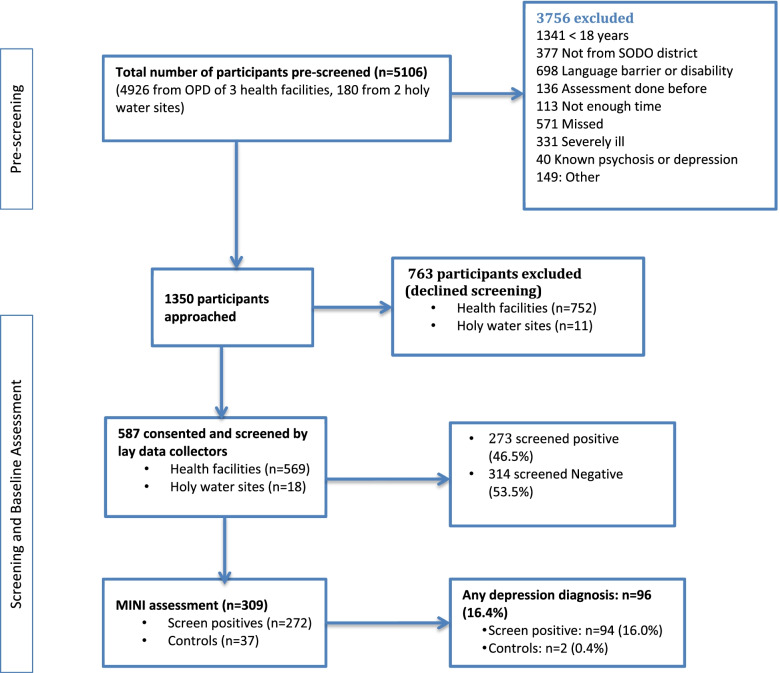
Participant recruitment flow chart

A total of 5106 consecutive patients attending three PHC facilities and two Holy water sites (places where religious treatment is being provided using Holy water) in the Sodo district were pre-screened (4926 from PHC facilities and 180 from holy water sites). Of these, 3756 were excluded due to several reasons (Fig. 1). Hence, 1350 were invited to participate in the study. Of these, 587 participants fulfilled the inclusion criteria and gave consent to be included in the study. Participant recruitment was done in two phases. In phase 1, 410 participants were recruited from 29 August 2019 to 17 March 2020; whereas 177 were recruited in phase 2 from 2 December 2020 to 18 February 2021. Patients were approached after they had consulted the PHC provider. Patients were recruited in to the study if they were adults (age ≥ 18), were able to speak and understand Amharic (the official language in Ethiopia), and gave informed consent.

### Brief screening questionnaires for depression

The brief depression screening tools considered in this study included the nine item Patient Health Questionnaire (PHQ-9) [30], the two item version of the patient health questionnaire (PHQ-2), the nine item PHQ with two additional contextually relevant items (PHQ-11), the Patient Health Questionnaire-15 (PHQ-15) [31] and the World Health Organization-Five Well-being Index (WHO-5) [32].

#### Patient health questionnaire (2-item, 9-item and 11 item versions)

The PHQ-9 is a nine item scale that was developed and validated as a depression assessment tool [30]. It has been widely used in clinical and population-based studies worldwide as a screening instrument [33]. The PHQ-9 follows the Diagnostic and Statistical Manual of Mental Disorders, 4th edition (DSM-IV) diagnostic criteria for a depressive episode [34] and asks about symptoms present in the preceding two weeks. Each question in the PHQ-9 is rated from 0 (not at all) to 3 (nearly every day). The response categories indicate the amount of time that the symptom was present. Responses for each item can be summed, which gives a total symptom score ranging from 0 to 27. The DSM diagnostic criteria for a major depressive episode can also be applied to give a possible diagnosis of depression using PHQ-9 [35]. The criterion validity of the PHQ-9 as a screening, but not diagnostic, tool has been demonstrated in Ethiopia in the PHC setting [20] and at a referral hospital in Addis Ababa [23]. A study was also conducted in Ethiopia on the criterion validity of the PHQ-9 among cancer patients attending the oncology clinic at a specialized hospital [24].

The PHQ-2 includes the first two items of the PHQ-9 and is usually used as an initial depression screening instrument for major depressive disorder [36]. The PHQ-2 has been found to be a useful screening tool in PHC settings, particularly in high-income countries [37]. Questions on the PHQ-2 have the same response categories as the PHQ-9 and ask about frequency of symptoms over the preceding two weeks. Responses on the PHQ-2 can be summed and scores range from zero to six. A study conducted in a PHC setting in Ethiopia showed that PHQ-2 has lower validity than the PHQ-9 [20]. The PHQ-11 includes all the items in the PHQ-9 and two new additional items (irritability and noise intolerance), which were found to be relevant expressions of depression in the Ethiopian context through qualitative studies and clinical experiences. A previous validation study in Ethiopia [20] found that 50% of the true cases of major depressive disorder had irritability.

#### Patient health questionnaire-15 (PHQ-15)

PHQ-15 is a brief, self-administered questionnaire that is used to assess somatic symptom severity and screen for the potential presence of somatisation and somatoform disorders based on the DSM-IV criteria in adults [31]. The PHQ-15 assesses the presence and severity of 15 common somatic symptoms in primary health care, such as fatigue in the last four weeks. The response categories for the PHQ-15 are “not bothered at all” (0), “bothered a little” (1) and “bothered a lot” (2) [38]. Aggregate scores range from 0 to 30 with higher scores indicating higher symptom severity. It has well-established psychometric properties, is available in multiple languages and has been recommended for use in large-scale studies [39].

In a recent systematic review [40], PHQ-15 had very high internal consistency, test-retest reliability, structural validity, and construct validity, as well as good criterion validity. Depression is less frequently diagnosed, particularly in non-Western settings, including in PHC, which may be due to somatization of depressive symptoms [41]. A somatic symptom-focused screening tool may be useful in detecting depression in these settings. Nevertheless, studies on the potential use of the PHQ-15 to screening depression in PHC are scarce. A study conducted among Korean College and graduate students showed that the PHQ-15 can be used as an effective screening tool for depression in that setting [42].

#### World Health Organization-Five Well-being Index (WHO-5)

The five item World Health Organization Well-Being Index (WHO-5) is a short and generic questionnaire which measures subjective well-being [32]. It consists of simple and non-invasive questions and the respondents are asked to rate how well each of the five statements apply to them over the last two weeks [43]. Each of the five items is scored from 5 (all of the time) to 0 (none of the time), with aggregate score ranging from 0 to 25. The WHO-5 has been translated into over 30 languages and has been used in research projects all over the world [44]. The tool is used widely for screening depression in the PHC setting [36].

A systematic review showed that the WHO-5 has adequate validity both as a screening tool for depression and as an outcome measure in clinical trials [44]; it has high criterion validity and is sensitive and specific as a screening tool for depression [43]. The review further found that the WHO-5 had a very high negative association with self- and observer-rated measures of depressive symptoms. However, the criterion validity of the WHO-5 has not been determined in the African setting. We found only one study which investigated the construct, discriminant and convergent validity of the scale in rural Kenya [45].

### Other measures

We used the seven item generalized anxiety disorder scale (GAD-7) to measure anxiety [46]. The GAD-7 has been validated both in PHC setting and the general population [47]. It measures how often the respondent has been bothered by seven symptoms of anxiety during the last two weeks with four response options ranging from 0 (not at all) to 3 (nearly every day). The scale has good sensitivity and specificity for the diagnosis of the most common anxiety disorders in primary care [48]. Overall, the GAD-7 is found to be a valid and efficient tool for detecting anxiety disorders [46]. The 12 item version of the World Health Organization Disability Assessment Schedule (WHODAS-2.0) [49] was used to assess functional impairment. The measure is considered to have cross-cultural validity [50] and the Amharic version has been validated in Ethiopia in a sample of people with severe mental disorders [51].

The Oslo social support scale (OSSS-3) was used to measure general social support. OSSS-3 is a three item scale which asks about number of close confidants, sense of concern or interest from other people and ease of getting practical help from neighbors [52]. OSSS-3 is a feasible instrument and has good predictive validity and convergent validity [53]. It has been used in previous community and facility-based studies in Ethiopia and demonstrated good utility [26]. The List of Threatening Experiences (LTE) was used to collect data on participants’ experience of stressful life events [54]. The LTE measures the occurrence of 12 prevalent major stressful events (e.g. death of a close relative or friend, loss of relationship, imprisonment and being the victim of theft) in the preceding 6 months, with dichotomous responses (yes/no). It has been adapted and used in a rural Ethiopian setting [26]. We developed and administered a structured self-report demographic and socio-economic characteristics questionnaire to collect data on such variables as sex, age, urban–rural residence, religion, marital status, educational status, and socio-economic status of the participants.

### Procedure

We used the Amharic version of the PHQ-9 which has already been adapted by Hanlon et al. [20]; the study also established the semantic, technical and content validity of the scale in the rural Ethiopian setting. The PHQ-15 and WHO-5 scales were translated into Amharic independently by two Ethiopian mental health researchers and then back-translated into English by two other researchers who are familiar with the study setting. We produced the final versions of these instruments through expert consensus. Changing the scales from self-completed to interviewer-administered required us to make some minor modifications as it has been done in previous studies from low-income countries [55].

Data for this study were collected by lay-data collectors and psychiatrists. Lay-data collectors administered the brief screening tools and other structured questionnaires. For those who obtained scores above the locally validated cut-off points in one of the depression screening scales and a random sample of screen negatives, clinical diagnosis of depression was done by Ethiopian psychiatrists. A semi-structured version of the depression module of the Mini-International Neuropsychiatric Interview (MINI) was used for clinical diagnosis by psychiatrists. The administration of the brief depression screening tools preceded the psychiatrist assessment. Psychiatrists were masked to the results of the screening questionnaires. All the lay-interviewers have at least diploma level training and have many years of experience in data collection related to mental health research. Senior mental health researchers trained the lay-data collectors for five days, which included role plays and observed pilot interviews. The psychiatrists who did the clinical assessment were trained by another senior Ethiopian psychiatrist for two days in the administration of the MINI. The training included role play and piloting of the clinical assessment.

### Data management and analysis

Data were checked for completeness in the field by research assistants and supervisors. Data were double entered with consistency checks in Epidata version 4.2.0. Data entry was done on the day of data collection where possible. Data cleaning was done using frequency distributions and logic checks, with reference to source documents as required. We analyzed the data using Stata version 14. Frequencies and percentages were used to summarize variables which were categorical, whereas continuous variables were summarized using mean and standard deviation.

We did exploratory factor analysis to investigate the construct validity of each of the brief depression screening questionnaires (except PHQ-2) with principal axis factoring method and we applied varimax rotation. Both Eigenvalues and scree plots were considered to determine the number of factors to be retained as indicators of scale dimensions. Rotated factor loadings were reported as indicators of the association of each item with the underlying factor. Concurrent and convergent validity were evaluated using non-parametric tests. Spearman’s rank order correlation coefficient (Rho) was computed for the association among the scores of each of the brief depression screening tools and scores of GAD-7, WHODAS-2, OSS-3 and LTE. The Mann Whitney test of the equality of medians was used to compare the distribution of each of the depression screening scale scores in people who have been diagnosed to have depression and in those who have not been diagnosed to have depression. Univariate logistic regression analysis was carried out to explore the association of each item in the depression screening tools with psychiatrist depression diagnosis. This was done to identify symptoms that are potentially useful to detect depression in the PHC setting. Internal consistency of items in each of the depression screening questionnaires was evaluated using Cronbach’s alpha.

### Ethical considerations

The study was conducted in accordance with the Declaration of Helsinki. The study protocol was reviewed and ethical approval was obtained from the Institutional Review Board of the College of Health Sciences, Addis Ababa University (Reference Number 007/18/Psy). Written informed consent was obtained from all the participants after the nature of the study and the information sought had been fully explained. Non-literate participants gave finger-prints to signify their willingness to participate. The nature and objective of the study were fully explained orally to the group of non-literate participants in understandable form and they gave finger-prints to signify their willingness to participate. Participants who were identified by the psychiatrists as having a mental health condition were provided with the appropriate treatment and follow-up in the out-patient psychiatric clinic in Bui Primary Hospital.

## Results

### Characteristics of study participants

We included a total of 587 adults into the study and all of these participants had complete data. A little more than half of the participants were men (51.3%). The mean age of the participants was 35.76 (SD = 13.8), and a quarter were not literate (26.1%). The majority of the participants were married (72.8%) and came from rural areas (69.1%). More than 95% of the participants were Christian. In terms of occupation, 38.7% were farmers and 26.2% were housewives. The main reason for their visit for the majority of the participants (74.1%) was new illness and a little more than a quarter of the participants (26.9%) had ever sought help in the facility or other places for their presenting complaint. See Table 1 for details of the socio-demographic characteristics of the participants.

**Table 1:** Socio-demographic characteristics of participants (*n* = 587)

Characteristics		N	%
Sex	Female	286	48.7
	Male	301	51.3
Age (years)	18–24	139	23.7
	25–34	159	27.1
	35–44	128	21.8
	45–64	133	22.7
	65+	28	4.8
		Mean 35.76	SD 13.8
Marital status	Never married	130	22.1
	Married	427	72.7
	Divorced	12	2.0
	Widowed	18	3.1
Residence	Urban	182	31.0
	Rural	405	69.0
Educational level	Cannot read and write	154	26.2
	Literate, but no formal education	77	13.1
	Primary school	205	34.9
	High school or above	152	25.9
Religion	Christian	559	95.2
	Muslim	28	4.8
Occupation	Paid work	62	10.6
	Private work	66	11.2
	Farming	226	38.5
	Housewife	154	26.2
	Student	53	9.0
	Unemployed	10	1.7
	Other	16	2.7
Income level or relative wealth	Very low	48	8.2
	Lower	195	33.2
	Average	321	54.7
	Higher	21	3.6
	Very high	2	0.3
Do you have children?	No	152	25.9
	Yes	435	74.1
Number of children (n = 435)	1–3 children	172	39.5
	4–6 children	192	44.1
	More than 6 children	71	16.3
What is the main reason for your visit today?	New illness	435	74.1
	Acute illness	39	6.6
	Injury	2	0.3
	Check-up or other preventive care	4	0.7
	Prenatal check-up	3	0.5
	Follow-up appointment for earlier chronic illness	94	16.0
	Follow-up appointment for earlier accident	3	0.5
	Other	7	1.2
Have you ever come to this facility or gone to other places for seeking help? (*n* = 584)	No	427	73.1
	Yes	157	26.9

*SD* Standard deviation, *n* Number of participants

### Construct validity

Exploratory factor analysis indicated, with both eigenvalue and scree plot criteria, that the PHQ-9, PHQ-11 and WHO-5 were unidimensional, with the factor in each scale explaining 51.0, 48.3 and 69.4% of the total variance, respectively. All items in the scales highly loaded onto the resulting factor. The factor loadings for the PHQ-9 ranged from 0.65 to 0.79; for PHQ-11 0.64 to 0.77; and for WHO-5 0.78 to 0.87. See Table 2.

**Table 2:** Rotated factor loadings of the exploratory factor analysis of PHQ-9, PHQ-11 and WHO-5

Item	Factor loading
PHQ-9	
Little interest or pleasure in doing things	0.74
Feeling down, depressed, or hopeless	0.79
Sleep problem	0.73
Feeling tired or having little energy	0.70
Eating problem	0.67
Feeling bad about yourself	0.74
Trouble concentrating	0.69
Agitation or retardation	0.72
Thoughts that you would be better off dead	0.65
PHQ-11	
Little interest or pleasure in doing things	0.72
Feeling down, depressed, or hopeless	0.77
Sleep problem	0.74
Feeling tired or having little energy	0.68
Eating problem	0.66
Feeling bad about yourself	0.73
Trouble concentrating	0.68
Agitation or retardation	0.69
Thoughts that you would be better off dead	0.64
Irritability	0.65
Noise intolerance	0.65
WHO-5	
Cheerful and good spirit	0.86
Calm and relaxed	0.87
Active vigorous	0.84
Fresh and rested	0.78
Interest	0.81

*WHO-5* World Health Organization-Five Well-being Index, *PHQ* Patient health questionnaire

The PHQ-15 was slightly different as there is a gender specific item (menstrual pain). First a factor analysis including all of the 15 items (PHQ-15a) resulted in three factors with eigenvalue >1. The first factor explained 30.8% and the other two factors explained 7.8 and 7.0% of the total variance. Most of the items clearly loaded onto their respective factors, with factor loadings ranging from 0.49 to 0.75 (Table 3). Two items (dizziness and fainting spells), cross-loaded onto two factors. It was generally very difficult to interpret the three factors as items loaded in each of the factors were mixed.

**Table 3:** Rotated factor loadings of the exploratory factor analysis of PHQ-15a and PHQ-15b

Item	Factor loading				
	PHQ-15a			PHQ-15b	
	Factor 1	Factor 2	Factor 3	Factor 1	Factor 2
Stomach pain		0.69			0.66
Back pain		0.54			0.61
Pain in your arms, legs, or joints	0.52				0.46
Menstrual cramps or other problems with periods			0.70		
Headaches		0.60			0.54
Chest pain	0.57			0.42	
Dizziness		0.42	0.45	0.55	
Fainting spells	0.48		0.52	0.71	
Heart pound or race	0.75			0.69	
Shortness of breath	0.70			0.68	
Pain or problems during sexual intercourse	0.49				
Constipation, loose bowels		0.61			0.60
Nausea		0.61			0.64
Tired or having low energy	0.61			0.64	
Trouble sleeping	0.47			0.46	

*PHQ-15* Patient health questionnaire-15

Factor analysis was performed again with the 13 items (PHQ-15b), excluding the gender specific item (Menstrual cramps or other problems with periods) and another item related to sexual intercourse (Pain or problems during sexual intercourse). The data for PHQ-15b (with the 13 items) seemed to suggest bifactorial structure. The first factor explained 34.1% and the second factor 8.5% of the total variance. Seven items loaded onto the first factor, with factor loadings ranging from 0.42 to 0.71, and six items loaded onto the second factor, with factor loadings from 0.54 to 0.66 (Table 4). There were no items which cross-loaded onto more than one factor. It appeared that items loaded onto the second factor were purely physical (e.g. stomach pain, back pain and problems with bowels); whereas factors loaded onto the first factor were not purely physical although they have physical manifestations (e.g. dizziness, trouble sleeping, tiredness, fainting and shortness of breath).

**Table 4:** Inter-correlation of depression screening scales and their correlation with anxiety, disability, social support and list of threatening events

		1	2	3	4	5	6	7	8	9
PHQ-2	(1)	1.00								
PHQ-9	(2)	0.87	1.00							
PHQ-11	(3)	0.85	0.98	1.00						
PHQ-15	(4)	0.61	0.74	0.75	1.00					
WHO-5	(5)	−0.58	−0.69	−0.70	−0.60	1.00				
GAD-7	(6)	0.63	0.71	0.73	0.64	−0.55	1.00			
WHODAS-2.0	(7)	0.65	0.74	0.74	0.69	−0.58	0.70	1.00		
OSSS-3	(8)	−0.31	−0.27	−0.27	−0.28	0.21	−0.33	−0.37	1.00	
LTE	(9)	0.38	0.40	0.42	0.37	−0.34	0.43	0.41	−0.27	1.00

*PHQ* Patient health questionnaire, *WHO-5* World Health Organization-Five Well-being Index, *GAD-7* seven item generalized anxiety disorder scale, *WHODAS* World Health Organization Disability Assessment Schedule, *OSSS-3* Oslo social support scale, *LTE* List of threatening experiences

Internal consistency of items, as evaluated by Cronbach’s alpha, for all the scales was good: 0.87 for PHQ-9, 0.89 for PHQ-11, 0.83 for PHQ-15 and 0.89 for WHO-5. Internal consistency for the PHQ-2 was lower (α = 0.72).

### Concurrent validity and convergent validity

Scores for all of the depression screening scales were moderately or highly correlated with each other (Rho = 0.58 to 0.98). The scores for PHQ-2, PHQ-9 and PHQ-11 were highly correlated (Rho = 0.85 to 0.98) with each other suggesting one could substitute the other. Scores for all the depression screening scales were moderately correlated with GAD-7 (Rho = 0.55 to 0.73) and WHODAS-2 scores (0.58 to 0.74). Scores for all of the depression screening scales were correlated with scores for social support and list of threatening events; however, the correlations were lower (Rho = 0.21 to 0.42). See Table 5. Differences between psychiatrist depression diagnosed and non-diagnosed cases in the median scores of all the screening scales were statistically significant (P < 0.01). The median score for PHQ-2, PHQ-9, PHQ-11, PHQ-15 and WHO-5 in the diagnosed cases were 3, 12, 15, 11 and 6, respectively; whereas in the non-diagnosed cases were 2, 7, 8, 8 and 9, respectively.

**Table 5:** Association of items in screening tools with depression diagnosis

Item	Odds Ratio (95% CI)	*P*-value
PHQ-9 + 2		
Little interest or pleasure in doing things	1.30 (1.05, 1.61)	<0.05
Feeling down, depressed, or hopeless	2.46 (1.87, 3.23)	<0.01
Sleep problem	2.03 (1.55, 2.67)	<0.01
Feeling tired or having little energy	1.63 (1.28, 2.08)	<0.01
Eating problem	1.40 (1.08, 1.81)	<0.01
Feeling bad about yourself	2.01 (1.56, 2.59)	<0.01
Trouble concentrating	1.97 (1.51, 2.57)	<0.01
Agitation or retardation	1.90 (1.43, 2.51)	<0.01
Thoughts that you would be better off dead	2.04 (1.49, 2.79)	<0.01
Irritability	2.64 (1.47, 4.72)	<0.01
Noise intolerance	2.25 (1.30, 3.91)	<0.01
PHQ-15		
Stomach pain	1.53 (1.10, 2.02)	<0.05
Back pain	1.17 (0.85, 1.61)	0.339
Pain in your arms, legs, or joints	1.50 (1.10, 2.05)	<0.05
Menstrual cramps or other problems with periods	1.31 (0.86, 1.99)	0.210
Headaches	1.53 (1.06, 2.20)	<0.05
Chest pain	1.39 (0.96, 2.02)	0.079
Dizziness	1.91 (1.32, 2.75)	<0.05
Fainting spells	2.20 (1.20, 4.04)	<0.05
heart pound or race	1.98 (1.39, 2.84)	<0.01
Shortness of breath	1.86 (1.30, 2.66)	<0.01
Pain or problems during sexual intercourse	2.08 (1.33, 3.24)	<0.01
Constipation, loose bowels	1.17 (0.80, 1.70)	0.423
Nausea, gas or indigestion	1.52 (1.10, 2.12)	<0.05
Feeling tired or having low energy	2.08 (1.41, 3.07)	<0.01
Trouble sleeping	2.26 (1.59, 3.23)	<0.01
WHO-5		
Cheerful and good spirit	0.60 (0.48, 0.75)	<0.01
Calm and relaxed	0.56 (0.45, 0.71)	<0.01
Active vigorous	0.52 (0.40, 0.67)	<0.01
Fresh and rested	0.698 (0.58, 0.85)	<0.01
Interest	0.58 (0.45, 0.74)	<0.01

*PHQ* Patient health questionnaire, *WHO-5* World Health Organization-Five Well-being Index, *CI* Confidence interval

### Association of ratings of items in screening tools with depression diagnosis

All of the PHQ-9 items were significantly associated with depression diagnosis (Table 5). However, items Feeling down, depressed or hopeless; Sleep problem; Feeling bad about oneself and Suicidal ideation were highly associated compared to the other items. The new items we added into the PHQ-9 (Irritability and Noise intolerance) had higher association than all of the PHQ-9 items, except the item Feeling down, depressed and hopeless. Although most of the PHQ-15 items had statistically significant association with depression diagnosis, the strength of association for most of the items was weak. Nevertheless, four items (Fainting spells, Problems during sexual intercourse, Feeling tired or having low energy and Trouble sleeping) had higher association compared to the other items. Overall, it appeared that items measuring emotional problems were highly associated with depression diagnosis more than items measuring cognitive or somatic symptoms. Particularly, items measuring physical problems had weak association with psychiatrist diagnosis of depression (Table 5). All items in the WHO-5 had negative and statistically significant association with depression diagnosis.

## Discussion

In this validation study of depression screening questionnaires in the PHC setting, PHQ-9, PHQ-11 and WHO-5 were found to be unidimensional and each item in all these scales highly loaded onto the resulting factor, suggesting that these instruments have good construct validity. A systematic review of the psychometric properties of the PHQ-9 [56] showed a good fit for both one factor and two factors solutions. Nevertheless, the one factor model is found to be more parsimonious. Exploratory factor analysis of the PHQ-9 in a PHC setting in rural Ethiopia [20] found that its internal structure is unidimensional, and all items loaded on to the resulting factor with item-factor correlation>0.35. The addition of two new items (irritability and noise intolerance) into the PHQ-9 did not change the structure of the scale, suggesting the utility and consistency of these items with the rest of the items. In the Ethiopian socio-cultural context, irritability and noise intolerance are strongly proscribed and usually considered as deviant states [20]. Several previous studies both from high-income countries and LMICs verified a one-factor structure of the WHO-5 through confirmatory factor analysis [44, 57]. A validation study of the Swahili version of the WHO-5 in rural coastal Kenya [45] found a unidimensional structure, and this was maintained across the three study groups (people living with epilepsy, people living with HIV and healthy controls).

PHQ-15 is found to be weak in terms of factor structure. The resulting factors when all items were considered are difficult to inteprete and some of the items cross loaded onto more than one factor. The original PHQ-15 validation study identified three factors when all of the items are considered [31]. However, there are also other studies that suggest bifactorial structure where there is a general somatic factor that all items load to as well as symptom-specific factors [58]. A number of studies, including the original PHQ-15 validation study, have excluded the item related to menstruation problem. In addition, this study has excluded another item (pain during sex) because it has low factor loading. There was similar observation in our data. As a result, the factor analysis was performed again with the 13 items. With the 13 items we found two factors where items clearly loaded onto their respective factors. Nevertheless, studies consistently show that the optimal structure of the PHQ-15 was bifactorial, providing both a single global measure of somatization and specific measures of pain, gastrointestinal, cardiopulmonary and fatigue factors [59].

All the depression screening questionnaires were strongly or moderately correlated with each other, suggesting concurrent validity. As expected PHQ-2, PhQ-9 and PHQ-11 were found to be correlated strongly with each other and moderately with the other depression screening questionnaires. PHQ-15 and WHO-5 were associated moderately with the other depression screening questionnaires. All of the depression screening scales were found to have moderate correlation with GAD-7 and WHODAS-2 and weak correlation (but in the expected direction) with social support and LTE, showing that they have good convergent validity. We found statistically significant difference in median scores of all the depression screening tools between psychiatrist diagnosed and non-diagnosed cases, suggesting known group validity of the depression screening questionnaires. This shows that all of the depression screening questionnaires considered in this study can discriminate between two groups known to differ in terms of depression diagnosis. Several previous studies show that both PHQ-9 and PHQ-2 have strong positive association with other depression screening questionnaires [36]. A systematic review study found that the WHO-5 has a very high negative association with self- and observer-administered measures of depressive symptoms [44]. Albeit depression symptoms and GAD-7 scores are different and independent, previous studies show that the two have moderate correlation [46, 48]. A validation study of both PHQ-9 and PHQ-2 in a PHC setting in rural Ethiopia showed that both are highly correlated with WHODAS 2.0 disability score and the number of days of disability in the preceding month [20].

We found that items measuring emotional problems were highly associated with depression diagnosis compared to items measuring somatic symptoms. Items measuring cognitive symptoms were in the middle. This is in contrast to the widely believed assertion that somatic symptoms are important to diagnose depression in non-western socio-cultural contexts. Several studies indicated that depression is least detected in non-western settings, including in PHC, because people in non-western cultures somatize depression [41]. Nevertheless, our study showed that patients who have depression are likely to report emotional and cognitive symptoms more than somatic symptoms. Clinicians are also likely to diagnose depression when patients report more of emotional and cognitive symptoms than somatic symptoms. A previous study conducted in a setting similar to ours [20] showed that although somatic symptoms were the most frequently endorsed symptoms in people with gold standard major depressive disorder, they were less discriminating than items that are more emotional and cognitive in nature. Overall, our study points that brief depression screening questionnaires in LMICs need to focus more on emotional and cognitive symptoms than somatic symptoms in order to increase detection of depression in the PHC setting. We also found that PHQ-15, which is a measure of severity of somatic symptoms [31], functions poorly compared to other measures in terms of construct validity, known group validity and predicting clinician diagnosis of depression. The two items we added to the PHQ-9 (irritability and noise intolerance) were found to be highly associated with clinician diagnosis of depression, suggesting their utility in screening depression in the PHC setting. A previous study pointed the potential utility of irritability as an important mood manifestation of depression in the Ethiopian socio-cultural setting [20]. The item “Little interest or pleasure in doing things” is found to be weak in prediciting diagnosis of depression. Hence, we suggest replacing this item with the two new items for screening purposes, but not for research, in the PHC setting in Ethiopia. The item “Feeling down, depressed, or hopeless” is highly associated with clinician diagnosis of depression, and we suggest including this item in the brief instrument to be used for screening depression in the PHC setting in Ethiopia.

Our study tried to investigate psychometric properties (concurrent and convergent validity, construct validity and known group validity) of depression screening questionnaires which were not emphasized in previous studies. The other strength of our study is that it recruited a large representative sample in the PHC setting. We are also able to report the psychometric properties of five depression screening questionnaires (PHQ-2, PHQ-9, PHQ-11, PHQ-15 and WHO-5). However, a few limitations of the study need to be highlighted. First, we did not determine the criterion validity (sensitivity, specificity, positive predictive values, negative predictive values and receiver operating characteristic curves) of the measures against a gold standard diagnosis of depression due to design constraints. The study was designed in such a way that clinician diagnosis of depression was done only for those who scored above the cut-off point in one of the depression screening questionnaires. Second, we were not able to evaluate the test-retest reliability and responsiveness to change of the measures due to feasibility constraints. Third, the study was facility-based and findings cannot be generalized into the general population. Lastly, we were unable to do confirmatory factor analysis as it was not possible to collect additional data from a separate sample due to feasibility constraints.

## Conclusions

The study demonstrates that the PHQ family of instruments, including the newly expanded version, and WHO-5 have good psychometric properties for use in the Ethipian PHC context. The two symptoms we added to the PHQ-9 are found to be useful to detect depression in the rural Ethiopian setting as they are highly associated with clinician diagnosis of depression. Inclusion of these symptoms also did not change the structure of the PHQ-9. Emotional and cognitive symptoms are found to be more useful than somatic symptoms to predict clinician diagnosis of depression. Hence, there is need to use depression screening scales with focus on emotional and cognitive symptoms to improve the detection of depression in the PHC setting. The study shows that the beliefe about the importance of somatic symptoms in recognizing depression and the high prevalence of these symptoms in patients seeking primary care for underlying depression in LMICs should be reexamined. Future research should focus on determining the criterion validity of PHQ-11 and WHO-5 and the test-retest reliability of all of the depression screening questionnaires considered in this study. Testing the unidimensional structure of PHQ-9, PHQ-11 and WHO-5 using confirmatory factor analysis is warranted.

## Data Availability

The datasets used and/or analyzed during the current study are part of an ongoing study [the IDEAS project] and cannot currently be publicly available [due to project requirements not to make the data publicly available until the forthcoming papers are drafted], but are available from the corresponding author [after the forthcoming papers are drafted] up on reasonable request.

## Abbreviations

DSM: Diagnostic and Statistical Manual of Mental Disorders
CI: Confidence interval
GAD-7: Seven item generalized anxiety disorder scale
IDEAS: Improving detection of depression in primary care in Sub-Saharan Africa
LMICs: Low and middle-income countries
LTE: List of threatening experiences
MINI: Mini-International Neuropsychiatric Interview
OSSS-3: Three item Oslo social support scale
PHC: Primary healthcare
PHQ: Patient health questionnaire
PRIME: Programme for Improving Mental Healthcare
Rho: Spearman’s rank order correlation coefficient
SD: Standard deviation
SNPPR: Southern Nations, Nationalities and peoples Region
WHO: World Health Organization
WHO-5: World Health Organization-Five Well-being Index
WHODAS: World Health Organization Disability Assessment Schedule
